# The comparison of water intake patterns and hydration biomarkers among young adults with different hydration statuses in Hebei, China

**DOI:** 10.1186/s12986-020-00531-2

**Published:** 2021-01-06

**Authors:** Jianfen Zhang, Na Zhang, Shufang Liu, Songming Du, Hairong He, Guansheng Ma

**Affiliations:** 1 Department of Nutrition and Food Hygiene, School of Public Health, Peking University, 38 Xue Yuan Road, Haidian District, Beijing, 100191 China; 2 Laboratory of Toxicological Research and Risk Assessment for Food Safety, Peking University, 38 Xue Yuan Road, Haidian District, Beijing, 100191 China; 3 School of Public Health, Hebei University Health Science Center, 342 Yuhua Road, Lianchi District, Baoding, 071000 China; 4 Chinese Nutrition Society, Room 1405, Beijing Broadcasting Building, No. 14 Jianguomenwai Street, Chaoyang District, Beijing, 100029 China

**Keywords:** Hydration biomarkers, Water intake patterns, Total water intake, Total drinking fluids, Water from food

## Abstract

**Background:**

Water is essential for maintaining the functions of human body properly. Studies have shown that the amounts and contributions of fluids were associated with health and hydration status. The objectives of the study was that to explore the differences of water intake pattern and hydration biomarkers among young males and females in different hydration statuses.

**Methods:**

A cross-sectional study was implemented among 159 young adults aged 18–23 years in Hebei, China. The total drinking fluids and water from food were obtained by 7-day 24-h fluid intake questionnaire and duplicate portion method, respectively. The osmolality and electrolyte concentrations of the 24 h urine and plasma were tested. Differences in optimal hydration (OH), middle hydration (MH) and hypohydration (HH) groups, divided by the osmolality of 24 h urine, were compared.

**Results:**

Totally, 156 participants (80 males and 76 females) completed the study. OH group had highest proportions of participants met the recommendations of total water intake (TWI) and total drinking fluids of China (34.5%, 36.2%), while HH group had lowest (7.7%, 0.0%). OH group had higher amounts of TWI, total drinking fluids, water and lower amounts of sugar-sweetened-beverages (SSBs) (*P* < 0.05). The percentage of total drinking fluids in TWI decreased from 54.1% in OH group to 42.6% in HH group (*P* < 0.05). OH group had higher and lower contributions of water and SSBs to total drinking fluids (*P* < 0.05); produced 551–950 mL more, excreted significantly less quantity of solutes of urine (*P* < 0.05). No significant differences were found in plasma osmolality among the three groups (*P* > 0.05). Among both males and females, the amounts of TWI and water were higher in OH group than others (*P* < 0.05). Males had 4.3% lower, 5.4% and 1.1% higher contributions of milk and milk products, SSBs and alcohol to total drinking fluids than females (*P* < 0.05); males had higher volume of urine than females only in MH group (*P* < 0.05). There were no significant differences of plasma osmolality between males and females in the same group (*P* > 0.05).

**Conclusions:**

Young adults with optimal hydration status had better water intake pattern and less concentrated urine. Females maybe have better water intake pattern than males.

*Trial registration* Chinese clinical trial registry. Name of the registry: Relationship of drinking water and urination. Trial registration number: ChiCTR-ROC-17010320. Date of registration: 01/04/2017. URL of trial registry record: http://www.chictr.org.cn/edit.aspx?pid=17601&htm=4.

## Background

As known, water is the main constitute of human body. It participates in many processes of metabolic activities of human beings, such as maintaining the balance of temperature and electrolyte of the body [1]. The balance between the input and output of the water defines the hydration status of humans. People will be in optimal hydration status when the input of the water is enough to make up for the output of the water, otherwise, if the input of the water is insufficient, people will be in hypohydration. Optimal hydration status is of vital importance for health. Downstream associations with low water intake and hypohydration were found in adults, such as increasing the risk of hyperglycemia [2], cardiovascular diseases and urinary system diseases [3]. Moreover, a series of studies have showed that hypohydration impeded the physical performances [4], cognitive performances [5] and mood [6].

Even though the recommendations of total drinking fluids had been proposed, there were still about 50% of the women and 60% of men failed to meet the recommendation of EFSA among adults in 13 countries [7]. Moreover, according to the results of fluids intake among people aged 18–60 years from four cities of China, approximately 32% of them had less amounts of total drinking fluids than the recommendation of China in 2007 [8], which may have adverse effects on their health. In a study conducted in the young male adults in China, about 1/4 of them did not drink enough to meet the total drinking fluids intake recommendation [9]. Moreover, in urban China, a relatively large proportion of people did not drink enough fluids [10]. It is urgent to take interventions to improve the amounts of total drinking fluids among people, in order to keep in optimal hydration status. Besides the amounts of total drinking fluids and the types of fluids were also worth being investigated. Researches showed that different types of fluids have different effects on health, such as the water, sugar-sweetened beverages (SSBs), etc. Among older women, the composition of total drinking fluids was associated with cardiovascular diseases [11]. Moreover, studies emphasized that SSBs could increase the risk of cardiovascular disease [12], obesity [13], hypertension [14] and type 2 diabetes [12]. However, substitution of plain water for SSBs was estimated to be associated with modestly lower risk of type 2 diabetes [15]. Therefore, it was important to investigate the amounts and types of different fluids, which was defined as drinking pattern.

The contributions of different fluids to total drinking fluids may vary between different countries, for example, water was the main contributor of total drinking fluid among adults, children and adolescents in China and Indonesia [10, 16], however, among Latin American adults, hot beverages predominated the total drinking fluids [17]. Studies also showed that the drinking patterns may associated with hydration status [18, 19]. But the drinking patterns among people in different hydration statuses were not investigated before, not to say the differences between different gender. In addition, in China, there was only one study showed that young males with optimal hydration status had higher water than those in hypohydration status [9], which needs more studies. It is important to elucidate the implications of differences of drinking patterns among participants with different hydration statuses. As known, the water from food was another main constitute of TWI. Because the content of water was different among different foods, the contributions of water from food to TWI varied among different countries, which from 19% in USA, 27% in UK [20] to 40% in China [8] or 51% in Japan [21]. Otherwise, the differences of the pattern of water from food including the amounts and contributions of different foods to water from food among participants in different hydration statuses were not explored, neither were the differences between different genders with the same hydration status.

Studies demonstrated that many indexes could evaluate the hydration statuses. Urinary biomarkers including the osmolality of 24 h urine [22], color [17, 23] and the void of urine [24] were showed to be sensitive to the changes of hydration statuses among people. A Study conducted among young women revealed that the osmolality was higher and the 24 h urine volume was lower in women with lower TWI than those with higher TWI [25]. Moreover, young male adults in China with hypohydration had more concentrated urine than those in optimal hydration status [9]. However, the differences among females with different hydration statuses, the differences of plasma biomarkers among participants with different hydration statuses and the differences between genders with the same hydration status were not demonstrated in China, which needs more studies.

The aims of the study were that, firstly, to investigate the differences in water intake patterns, including the drinking patterns and the patterns of water from food among participants in different hydration statuses, both in males and females; secondly, to explore the differences of hydration biomarkers including urinary and plasma biomarkers among participants in different hydration statuses; thirdly, to find out the differences in the patterns of water intake and hydration biomarkers between males and females with the same hydration status.

## Materials and methods

### Study design

This was a cross-sectional study on water intake patterns and hydration biomarkers which lasted for 7 days.

### Sample size calculation

According to a research performed among young male adults [9], the total drinking fluids were 1733, 1250, 936 mL, and the standard deviation were 399, 342, 281, respectively, among the participants from the optimal hydration, middle hydration and hypohydration groups. Then, software PASS 11.0 (NCSS, LLC, Kaysville, UT) was used to calculate the sample size for the differences of the water intake patterns and hydration biomarkers among participants with different hydration statuses. Statistical significance α was set at 0.05 (*P* < 0.05, 2-tailed), power (1-Beta) was 0.90, k (number of groups) was 3. In addition, 20% drop-out rate was considered. Eventually, 36 participants were needed in this study, with 12 participants in every group.

However, at the third day of the 7 days, three females were excluded. One volunteer quit because she could not cooperate with the study and the other two were slightly discomfort.

### Participants

Participants were selected form a university in Hebei, China.

The inclusion criteria: participants included healthy males and females, aged 18–23 years were included.

The exclusion criteria: participants aged < 18 years or > 23 years, or participants with smoking, habitual alcohol consumptions (> 20 g/day) or habitual high caffeine consumptions (> 250 mg/day) or had chronic diseases or other diseases were excluded from the study [26].

### Ethics approval

The study protocol was approved by the Peking University Institutional Review Board. The ethical approval project identification code was IRB00001052-16071. The study was conducted according to the guidelines of the Declaration of Helsinki. Written informed consent was obtained from all participants before they participated in the study.

### Study procedure

**Fig. 1:**
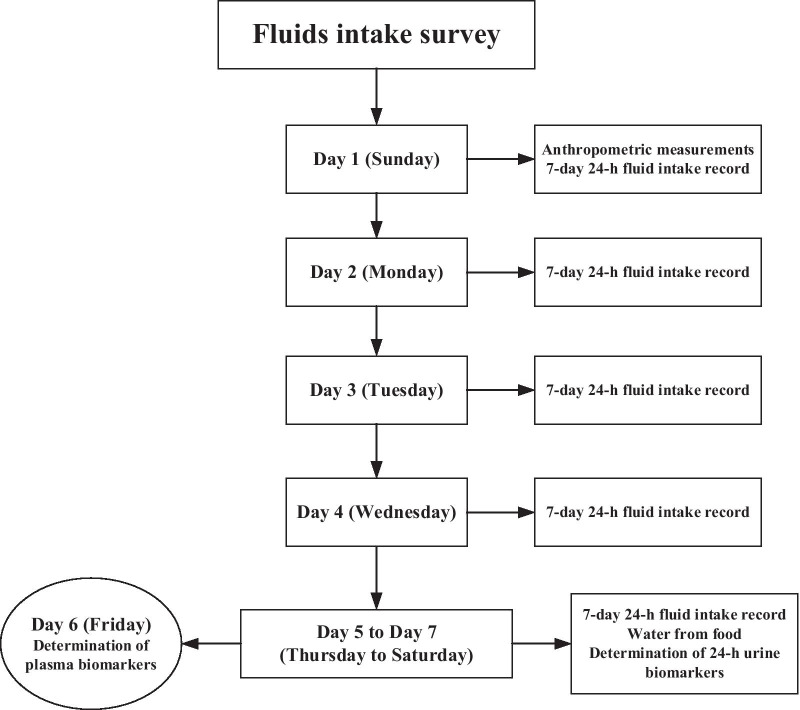
The study procedure

A cross-sectional study was designed and conducted. The study period included 7 consecutive days. Participants were asked to complete the 7-day 24-h fluid intake questionnaire for 7 consecutive days. On the first day of the study, the anthropometric measurements including height and weight were performed. All participants were instructed to use the 7-day 24-h fluid intake questionnaire designed by the researchers to record the total drinking fluids. From the first day to the seventh day of the study, participants were asked to record the total drinking fluids as usual. The amount of fluid intake for each time was measured by a cup with the nearest of 5 mL. On the fifth day to the seventh day of the study, participants were asked to collect every urine and all the food they ate were weighed and recorded during the 3 days. On the sixth day, the fasting blood samples of all participants were collected. The temperature and humidity of indoor and outdoor was recorded at 10:00 a.m., 2:00 p.m. and 8:00 p.m. each day for 7 days. The study procedure was shown in Fig. 1.

### Definition

Total water intake (TWI) = Total drinking fluids + Water from food. Total drinking fluids included the amount of fluids as follow [26]: (1) water, including plain water, tap water and bottle water; (2) tea, including fermented tea and semi-fermented tea; (3) milk and milk products, including liquid milk, yogurt and other milk products; (4) SSBs, including carbonated drinks, sports drinks, sweetened fruit juice and vegetable juice and other sugared drinks; (5) alcohols, including wine, beer, liquor and other alcoholic beverages; (6) other beverages.

Water from food included the intake of water from food as follow [26]: (1) staple food, including steamed bread and rice; (2) dishes, including vegetables, meat and eggs; (3) porridge, including millet porridge and other porridges; (4) soup, including tomato egg soup and other soups; (5) snacks, including fruits and other snacks.

Patterns of water intake = Drinking pattern + Pattern of water from food. Drinking Pattern, included the amounts of total drinking fluids, the amounts of different fluids and the contributions of different fluids to total drinking fluids. Pattern of water from food, included the amounts of water from food, the amounts of water from different food and contributions of different food to water from food.

Hydration status was defined according to the osmolality of 24 h urine. The optimal hydration is defined as urine osmolality ≤ 500 mOsm/kg, middle hydration is defined as 500 mOsm/kg<urine osmolality ≤ 800 mOsm/kg, hypohydration is defined as urine osmolality > 800 mOsm/kg [9, 22]. Therefore, participants were divided into three groups on the basis of their osmolality of 24 h urine mentioned above, i.e. the OH (optimal hydration) group, MH (middle hydration) group and the HH (hypohydration) group.

### Anthropometric measurements

Height and weight were measured twice by trained investigators in standard procedure (HDM-300; Huaju, Zhejiang, China). [BMI: weight (kg)/height squared (m^2^)]. The values of height and weight were showed as the calculated averages, respectively.

### Temperature and humidity of the environment

The temperature and humidity both indoors and outdoors was recorded by researcher each day using a temperature hygrometer (WSB-1-H2, Exasace, Zhengzhou, China). The times that the researcher recorded every day during the 7 days were that at 10:00 a.m., 2:00 p.m. and 8:00 p.m.

### Assessment of total water intake

#### Assessment of total drinking fluids

A self-designed 7-day 24-h fluid intake record questionnaire was used to assess the total drinking fluids. The type and amount of fluids for each time was measured by a cup to the nearest of 5 mL.

#### Assessment of water from food

Water from food was assessed with duplicate portion method. The samples of food being weighed before and after participants ate and the backup food samples collected for three consecutive days. All foods were weighed accurately by trained investigators using electronic balance (YP20001; SPC; Shanghai, China). Moreover, the backup food samples were stored in refrigerators at + 4 °C and sent to laboratory to be measured within 36 h. The samples of foods were measured according to National Food Safety Standard GB 5009.3-2016 Determination of water in Food [27] by laboratory analyst in Beijing Institute Nutritional Resource. Parallel samples were taken for each food samples, and the error between the two results was no more than 5%. Water intake from fruits was assessed using the China Food Composition Table (2009) [28].

#### Determination of urine biomarkers

The 24 h urine was defined from the second urine of the first day to the first urine of the second day. The 24 h urine samples of three consecutive days were collected by participants using self-designed containers of the investigators. All the urine samples were stored at + 4 °C before measured. Every urine sample was collected and tested within 2 h. Urine volume was measured to the nearest 0.1 g using a desktop electronic scale (YP20001, SPC, Shanghai, China). Urine osmolality was assessed with freezing point method by osmotic pressure molar concentration meter (SMC 30C; Tianhe, Tianjin, China). USG (urine specific gravity) and pH were tested by automatic urinary sediment analyzer with uric dry-chemistry method (H-800; Dirui, Changchun, China). Urine electrolyte concentrations (including Na, K, Cl, Ca, Mg and phosphate), urine acid, urine urea nitrogen and creatinine were tested by automatic biochemical analyzer with the ion-selective electrode potentiometer method (AU 5800; Beckman, Brea, CA, USA).

In order to make sure that all the urines were collected, we took four measures to improve quality of the control. Firstly, participants were asked to record the information of every urine, including the time and the voids, on the questionnaire of “3-day-24-h urinary behavior questionnaire”. They also should write the same information on the urine container. Moreover, they should give the questionnaire to the investigators every day. Secondly, when the samples of urine were sent to the laboratory, the researchers were asked to record the information of the urine, including the time and the voids into the questionnaire that designed for them. Thirdly, researchers should compare the two records to find if there were some differences. If there were differences, researchers should check the questionnaire and the information on the urine container, to correct the errors. Fourthly, we also compared the information of the urine and the information of total drinking fluids to make sure that all the samples of urine were recorded.

#### Determination of plasma biomarkers

Fasting blood samples were collected for 1 day to measure the osmolality and electrolyte concentrations. Plasma osmolality was assessed with freezing point method by osmotic pressure molar concentration meter (SMC 30C; Tianhe, Tianjin, China). Blood electrolyte concentrations (including sodium, potassium, chloride, calcium, magnesium and phosphate) were tested by automatic biochemical analyzer with the ion-selective electrode potentiometer method (AU 5800; Beckman, Brea, CA, USA).

#### Statistics

The SAS 9.2 software (SAS Institute Inc., Cary, NC, USA) was used for statistical analysis. Mean ± standard deviation (SD) was used to present data with normal distribution. While, median and quartile ranges (Q) was used to describe data in skewness distribution. The TWI of participants in the three groups were compared to the recommendation of WHO, EFSA and China, respectively. The recommendations of total water intake in WHO, EFSA and China for males and females were, 2.9 L/day, 2.2 L/day; 2.5 L/day, 2.0 L/day and 3.0 L/day, 2.7 L/day, respectively. One-way ANOVA, Kruskal–Wallis H test, Mann–Whitney U test and Student’s *t* test were used to compare the differences among the three groups, among the same gender in the three groups and between different genders in the same group. Spearman’s correlation coefficients were performed to determine the strength of the relationship between amounts of food intake, amounts of water from food and the contributions of water from food to TWI. Significance levels were set at 0.05 (*P* < 0.05).

## Results

A total of 159 participants were recruited for the study, and 156 of them completed the study, with a 98% completion rate, including 80 males and 79 females. The proportions of participants in OH, MH and HH groups were 37.1%, 46.2%, and 16.7%, respectively. Significant differences were found in height, weight and BMI among participants in three groups (*P *< 0.05). Among males in the three groups, significant differences were found in height, weight and BMI (*P *< 0.05), but no significant differences were found among females in the three groups (*P > *0.05). Between males and females in the same group, differences were found in the height and weight (*P *< 0.05), as shown in Table 1.

**Table 1:** The characteristics of participants

	OH (n = 58)			MH (n = 72)			HH (n = 26)		
	Male (n = 19)	Female (n = 39)	Total (n = 58)	Male (n = 44)	Female (n = 28)	Total (n = 72)	Male (n = 17)	Female (n = 9)	Total (n = 26)
Age (year)	20.0 ± 1.0	19.9 ± 1.0	19.9 ± 1.0	19.7 ± 1.0	19.8 ± 1.3	19.7 ± 1.1	20.1 ± 1.0	19.1 ± 0.8	19.8 ± 1.1
Height (cm)	171.1 ± 6.9	159.5 ± 5.4	163.3 ± 8.0*	173.2 ± 4.8	161.3 ± 7.1	168.6 ± 8.2	169.8 ± 4.7	159.2 ± 5.9	166.1 ± 7.2^‡^
Weight (kg)	64.1 ± 8.8	53.4 ± 6.6	56.9 ± 8.9*	69.6 ± 11.9	56.6 ± 6.4	64.6 ± 12.0	66.0 ± 14.0	55.5 ± 6.6	62.3 ± 12.8^‡^
BMI (kg/m^2^)	21.9 ± 2.6	21.0 ± 2.2	21.3 ± 2.3*	23.2 ± 4.1	21.8 ± 2.3	22.7 ± 3.6	22.8 ± 4.5	21.9 ± 2.3	22.5 ± 3.8^‡^

Values are shown as the mean ± standard deviation (SD)

*There was statistically significant difference between OH and MH groups, *P* < 0.05

^†^There was statistically significant difference between MH and HH groups, *P* < 0.05

^‡^There was statistically significant difference between OH and HH groups, *P* < 0.05. One-way ANOVA was used to compare the differences among the three groups, both in males and females. Significant differences were found in height, weight and BMI among subjects in the three groups (*F* = 6.921, *P* = 0.001; *F* = 7.834, *P* = 0.001; *F* = 3.361, *P* = 0.037). Significant differences were found in height, weight and BMI in males among the three groups (*F* = 6.921, *P* = 0.001; *F* = 7.834, *P* = 0.001; *F* = 3.361, *P* = 0.037); no significant differences were found in females among the three groups (*P > *0.05)

### Temperature and humidity

The average indoor and outdoor temperature for the 7 days was 21.8 °C and 20.7 °C, respectively. The average indoor and outdoor humidity was 39.9% and 35.9%, respectively, see Table 2.

**Table 2:** The temperature and humidity of study days

	Indoors		Outdoors	
	Temperature (°C)	Humidity (%)	Temperature (°C)	Humidity (%)
Sunday	19.9	43	18.1	37
Monday	23.0	48	22.4	41
Tuesday	23.3	31	24.0	29
Wednesday	21.5	48	17.9	42
Thursday	21.5	40	21.0	36
Friday	22.2	35	19.2	35
Saturday	21.2	34	22.6	31

### Comparison of the water intake pattern

It was demonstrated in Table 3 that the amounts of food intake, staple food, dishes, porridge did not differ significantly among the three groups (all *P > *0.05), while, the intakes of soup were significantly different (*P *< 0.05). The intake of soup was higher in OH group than MH group and HH group (*P* = 0.012; *P* = 0.023), and no significant differences were found in the intake of soup between MH group and HH group (*P* = 1.000). Among males in the three groups, the amounts of the intakes of food, staple food, dishes, soup and porridge did not differ significantly (all *P > *0.05). Interestingly, among females, there were no significant differences were found in the amounts of staple food, dishes and soup among the three groups in females (all *P > *0.05). While, the amounts of the food intake and porridge were significantly different among the three groups in females (*P* = 0.010; *P* = 0.008). For the amounts of food intake, OH group was higher than that of HH group (*P* = 0.029); MH group was higher than that of HH group (*P* = 0.029), and no significant differences were found between OH and MH groups (*P* = 1.000). In the intake of porridge, OH group was higher than that of MH group (*P* = 0.029), but with no significant differences with MH group (*P* = 0.181); MH group did not differ significantly with HH group (*P* = 0.872). The relationships showed that with the increase of the intake of food, the amounts of water from food increased (r = 0.965, *P *< 0.001). While, the relationship between the amounts of the intake of food and the percentages of water from food in TWI was not significantly (r = 0.141, *P* = 0.079).

**Table 3:** Food intake of participants

	OH (n = 58)			MH (n = 72)			HH (n = 26)		
	Male (n = 19)	Female (n = 39)	Total (n = 58)	Male (n = 44)	Female (n = 28)	Total (n = 72)	Male (n = 17)	Female (n = 9)	Total (n = 26)
Food intake (g)	1735 (420)	1395 (411)	1537 (510)	1814 (481)	1307 (356)	1588 (544)	1710 (651)	1099 (150)	1357 (788)
Staple food (g)	686 (252)	436 (164)	514 (246)	696 (161)	464 (214)	606 (254)	649 (251)	383 (104)	530 (275)
Dishes (g)	852 (243)	627 (275)	736 (284)	840 (278)	668 (245)	759 (228)	761 (321)	463 (271)	724 (376)
Porridge (g)	129 (169)	194 (228)	160 (222)	95 (138)	107 (169)	105 (134)	59 (197)	49 (95)	52 (161)
Soup (g)	66 (192)	73 (193)	73 (192)	179 (239)	93 (171)	116 (211)	118 (259)	110 (298)	114 (284)

Values are shown as the median (M) and quartile ranges (Q). The differences of the amounts of food intake were compared using the method of Kruskal–Wallis *H* test among the three groups, both in males and females

The amounts of food intake, staple food, dishes, porridge did not differ significantly among the three groups (*χ*^2^ = 3.223, *P* = 0.200; *χ*^2^ = 5.319, *P* = 0.070; *χ*^2^ = 4.540, *P* = 0.103; *χ*^2^ = 4.030, *P* = 0.133), while, the intakes of soup were significantly (*χ*^2^ = 10.891, *P* = 0.004). When compared the three groups with each other, the intake of soup was higher in OH group than MH group and HH group (*χ*^2^ = 22.915, *P* = 0.012; *χ*^2^ = 28.406, *P* = 0.023), and no significant differences were found in the intake of soup between MH group and HH group (*χ*^2^ = 5.492, *P* = 1.000). There were no significant differences were found in the amounts of food intake, staple food, dishes, porridge and soup among the three groups in males (*χ*^2^ = 0.877, *P* = 0.645; *χ*^2^ = 1.688, *P* = 0.430; *χ*^2^ = 2.658, *P* = 0.265; *χ*^2^ = 1.311, *P* = 0.519; *χ*^2^ = 3.976, *P* = 0.137). There were no significant differences were found in the amounts of staple food, dishes and soup among the three groups in females (*χ*^2^ = 2.727, *P* = 0.256; *χ*^2^ = 4.332, *P* = 0.115; *χ*^2^ = 0.722, *P* = 0.697). The amounts of the food intake and porridge were significantly different among the three groups in females (*χ*^2^ = 9.219, *P* = 0.010; *χ*^2^ = 9.677, *P* = 0.008). In the amounts of the intake of food, the intake of OH group was higher than that of HH group (*χ*^2^ = 6.701, *P* = 0.029); MH group was higher than that of HH group (*χ*^2^ = 6.708, *P* = 0.029), and no significant differences were found between OH and MH groups (*χ*^2^ = 0.787, *P* = 1.000). In the intake of porridge, OH group was higher than that of MH group (*χ*^2^ = 6.701, *P* = 0.029), but with no significant differences with MH group (*χ*^2^ = 3.528, *P* = 0.181); MH group did not differ significantly with HH group (*χ*^2^ = 1.117, *P* = 0.872)

As shown in Table 4, there were significantly 43.6–52.6%, 32.8–64.4% and 22.0–26.8% more participants in OH group met the recommendations of TWI of WHO, EFSA and China, respectively (*P *< 0.001; *P *< 0.001; *P* = 0.002), and 25.1–36.2% more participants met total drinking fluids according to the reference of China than that in MH and HH groups (*P *< 0.001). The results revealed significant between-group differences in TWI, total drinking fluids among the three groups, with OH group consuming 2504 mL, 1362 mL and HH group consuming 1839 mL and 913 mL, respectively (*P *< 0.001; *P *< 0.001). The amounts of water from food were similar among the three groups (*P > *0.05). According to the amounts of different fluids and water from foods, OH group had the highest water intake and water from porridge (*P *< 0.001; *P* = 0.002; *P* = 0.001). Moreover, 6–66 mL and 0 mL lower amounts of SSBs and alcohols were found in OH group than that in MH and HH groups (*P* = 0.013; *P* = 0.020), respectively. Both in males and females, OH group had higher amounts of TWI, total fluids intake and water and lower SSBs than those in other groups (*P *< 0.05). In males, the intakes of water from food were similar among the three groups but in females, OH group was the highest among the three groups (*P *< 0.05). Among the three groups, males had higher amounts of TWI and water from food than females in the same group (*P *< 0.05). In MH and HH groups, males had higher amounts of total drinking fluids than that of females in the same group (*P *< 0.05).

**Table 4:** Fluids intake of participants

		OH (n = 58)						MH (n = 72)						HH (n = 26)					
		Male (n = 19)		Female (n = 39)		Total (n = 58)		Male (n = 44)		Female (n = 28)		Total (n = 72)		Male (n = 17)		Female (n = 9)		Total (n = 26)	
		M (Q)	%	M (Q)	%	M (Q)	%	M (Q)	%	M (Q)	%	M (Q)	%	M (Q)	%	M (Q)	%	M (Q)	%
Total water intake (TFI)		2893 (1052)		2443 (625)		2504 (1288)*		2554 (674)		1855 (408)^†^		2192 (791)^†^		2133 (654)		1418 (373)		1839 (914) ^‡^	
Percentage meeting adequate TFI of China (%)		8 (42.1%)		12 (30.8%)		20 (34.5%)*		7 (15.9%)		2 (7.1%)		9 (12.5%)		2 (11.8%)		0 (0.0%)		2 (7.7%)^‡^	
Percentage meeting adequate TFI of EFSA (%)		12 (63.2%)*		32 (82.1%)*		44 (75.9%)*		24 (54.5%)*		7 (25.0%)^†^		31 (43.1%)		3 (17.6%)		0 (0.0%)^‡^		3 (11.5%)^‡^	
Percentage meeting adequate TFI of WHO (%)		9 (47.4%)		26 (66.7%)*		35 (60.3%)*		8 (18.2%)		4 (14.3%)		12 (16.7%)		2 (11.8%)		0 (0.0%)^‡^		2 (7.7%)^‡^	
Total drinking fluids (mL)		1548 (909)	54.4%	1271 (774)	53.9%	1362 (799) *	54.1%*	1220 (484)	48.6%	785 (342)	45.4%	1060 (588) ^†^	47.4%^†^	967 (273)	45.3%	521 (226)	37.6%	913 (522)^‡^	42.6%^‡^
Percentage meeting adequate total drinking fluids of China (%)		9 (47.4%)*		12 (30.8%)		21 (36.2%)*		6 (13.6%)		2 (7.1%)		8 (11.1%)^†^		0 (0.0%)^†^		0 (0.0%)		0 (0.0%)^‡^	
Water		1532 (1016)	85.3%	1053 (707)	84.2%	1194 (867) *	84.6% *	971 (463)	81.6%	698 (216)^†^	77.8%^†^	812 (475)	80.2%	597 (498)	68.2%	226 (204)	54.6%	418 (461)^‡^	63.5%^‡^
Tea		0 (0)	0.0%	0 (0)	0.7%	0 (0)	0.5%	0 (0)	0.3%	0 (0)	4.5%	0 (0)	1.9%	0 (0)	0.6%	0 (0)	0.0%	0 (0)	0.4%
Milk and milk products		29 (114)	4.5%	79 (141)	7.6%	67 (141)	6.6%	20 (141)	5.1%	65 (151)	9.4%	40 (141)	6.8%	0 (110)	7.1%	43 (88)	11.7%	7 (97)	8.7%
SSBs		50 (83)	8.5%	9 (76)	3.9%	33 (80) *	5.4%*	47 (217)	10.2%	0 (55)	4.8%	38 (123)	8.1%	86 (352)	19.1%	112 (201)	25.7%	99 (301)^‡^	21.4%^‡^
Alcohol		0 (0)	0.0%	0 (0)	0.0%	0 (0) *	0.0%*	0 (0)	1.1%	0 (0)	0.0%	0 (0)	0.7%	0 (18)	2.7%	0 (0)	0.0%	0 (0)^‡^	1.8%^‡^
Others		0 (36)	1.6%	4 (40)	3.4%	0 (38)	2.8%	2 (32)	1.6%	11 (36)	3.6%	9 (32)	2.4%	0 (23)	2.3%	36 (60)	8.0%	2 (50)	4.3%
Water from food (mL)		1312 (323)	45.6%	1088 (336)	46.1%	1183 (414)	45.9%*	1299 (323)	51.4%	1001 (259)	54.6%	1184 (358)	52.6%	1241 (467)	54.7%	798 (149)	62.4%	999 (549)	57.4%^‡^
Staple food		385 (120)	30.0%	252 (104)	22.1%	280 (160)	24.7%	369 (120)	29.2%	265 (159)	24.3%	331 (129)	27.3%	329 (127)	28.6%	210 (82)	25.3%	268 (143)	27.5%
Dishes		697 (217)	53.0%	559 (211)	50.1%	620 (227)	51.0%	688 (216)	53.1%	548 (219)	54.7%	632 (190)	53.7%	624 (237)	51.1%	383 (253)	51.7%	592 (313)	51.3%
Soup		62 (183)	5.9%	69 (183)	16.8%	69 (183)	7.7%	170 (227)	10.9%	88 (163)	9.8%	105 (201)	10.4%	113 (246)	11.9%	46 (105)	15.6%	108 (264)	13.2%
Porridge		95 (157)	9.9%	175 (218)	8.7%	149 (222)*	14.5%*	78 (140)	6.8%	98 (176)^†^	10.4%	87 (144)	8.2%^†^	43 (195)	8.2%	104 (271)	7.4%	45 (167)^‡^	7.9%^‡^
Snacks		0 (5)	1.3%	0 (40)	2.4%	0 (32)*	2.0%*	0 (0)	0.1%	0 (0)	0.9%	0 (0)	0.4%^†^	0 (0)	0.2%	0 (0)	0.0%	0 (0)^‡^	0.2%^‡^

Values are shown as the median (M) and quartile ranges (Q)

According to the references of TWI in EFSA, WHO and China, there were significantly more subjects in OH group had enough TWI than that in MH and HH groups (*χ*^*2*^ = 32.291, *P *< 0.001; *χ*^*2*^ = 36.592, *P *< 0.001; *χ*^*2*^ = 12.656, *P* = 0.002). In males, there were significantly more subjects in OH group had enough TWI than that in MH and HH groups (*χ*^*2*^ = 7.072, *P* = 0.025; *χ*^*2*^ = 8.753, *P* = 0.013; *χ*^*2*^ = 5.823, *P* = 0.058) In females, there were significantly more subjects in OH group had enough TWI than that in MH and HH groups (*χ*^*2*^ = 26.056, *P *< 0.001; *χ*^*2*^ = 33.687, *P *< 0.001; *χ*^*2*^ = 7.536, *P* = 0.020). According to the reference of total drinking fluids in China, there were significantly more subjects in OH group had enough total drinking fluids than that in MH and HH groups, in total, males and females, respectively (*χ*^*2*^ = 20.476, *P *< 0.001; *χ*^*2*^ = 13.363, *P* = 0.001; *χ*^*2*^ = 7.536, *P* = 0.020)

Significantly differences were found in the contributions of total drinking fluids and water from food to TWI among the three groups (*F* = 18.726, *P *< 0.001; *F* = 18.726, *P *< 0.001). Refer to the percentages of fluids to total drinking fluids, there were significant differences in the percentages in the amounts of water, SSBs and alcohols in total drinking fluids (*F* = 13.715, *P *< 0.001; *F* = 13.912, *P *< 0.001; *F* = 3.956, *P* = 0.021). There were significant differences of the contributions of water from porridge and snacks to water from food (*F* = 8.349, *P *< 0.001; *F* = 5.686, *P* = 0.004). In males, significantly differences were found in the contributions of total drinking fluids and water from food to TWI among the three groups (*F* = 6.116, *P* = 0.003; *F* = 6.116, *P* = 0.003). In males, there were different percentages of the intake of water and water from snacks in water from food (*F* = 4.473, *P* = 0.015; *F* = 5.710, *P* = 0.005). In females, significant differences were found in the percentage of total drinking fluid and water from food in TWI (*F* = 15.951, *P *< 0.001; *F* = 15.951, *P *< 0.001); according to the contributions of different fluids to total drinking fluids, differences were found in water, SSBs and water from porridge (*F* = 12.877, *P *< 0.001; *F* = 17.594, *P *< 0.001; *F* = 5.092, *P* = 0.009)

Differences were found in the amounts of TWI, total drinking fluids, water, SSBs, alcohols and the water from porridge and snacks among the three groups (*χ*^2^ = 23.421, *P *< 0.001; *χ*^2^ = 31.087, *P* = 0.013; *χ*^2^ = 40.126, *P *< 0.001; *χ*^2^ = 8.654, *P* = 0.013; *χ*^2^ = 7.830, *P* = 0.020; *χ*^2^ = 12.639, *P* = 0.002; *χ*^2^ = 14.303, *P* = 0.001), but no significant differences were found in the intake of water from food (*χ*^2^ = 4.010, *P* = 0.135). There were differences in the amounts of TWI, water from food, and water from staple food, dishes between males and females in OH group (*Z* = − 2.444, *P* = 0.015; *Z* = − 2.841, *P* = 0.004; *Z* = − 4.664, *P* = 0.000; *Z* = − 3.405, *P* = 0.001). In males, differences were found in the amounts of TWI, total drinking fluids, water and water from snacks (*χ*^2^ = 16.361, *P *< 0.001; *χ*^2^ = 18.832, *P *< 0.001; *χ*^2^ = 11.524 *P* = 0.003). In females, differences were found in the amounts of TWI, total drinking fluids, water from food, water, SSBs and water from porridge (*χ*^2^ = 35.127, *P *< 0.001; *χ*^2^ = 36.403, *P *< 0.001; *χ*^2^ = 13.549, *P* = 0.001; *χ*^2^ = 37.029, *P *< 0.001; *χ*^2^ = 7.466, *P* = 0.024; *χ*^2^ = 9.745, *P* = 0.008). In the MH group, there were significant differences in the amounts of TWI, total drinking fluids, water from food, water, SSBs, alcohol and water from staple food, dishes and snacks (*Z* = − 5.117, *P *< 0.001; *Z* = − 4.147, *P *< 0.001; *Z* = − 4.713, *P *< 0.001; *Z* = − 4.292, *P *< 0.001; Z = − 2.685, *P* = 0.007; Z = − 2.024, *P* = 0.043; Z = − 5.002, *P *< 0.001; Z = − 3.523, *P *< 0.001; Z = − 1.966, *P* = 0.049). In the HH group, males had higher amounts of TWI, total drinking fluids, water from food, water, water from staple food and dishes than females (*Z* = − 3.800, *P* = 0.000; *Z* = − 3.476, *P* = 0.001; *Z* = − 2.937, *P* = 0.001; *Z* = − 3.369, *P* = 0.000; *Z* = − 3.422, *P* = 0.001; *Z* = − 2.345, *P* = 0.019)

*There was statistically significant difference between OH and MH groups, *P* < 0.05

^†^There was statistically significant difference between MH and HH groups, *P* < 0.05

^‡^There was statistically significant difference between OH and HH groups, *P* < 0.05. The One-way ANOVA and Kruskal–Wallis *H* test were used to compare the differences among the three groups, including males and females. When comparing the differences between males and females within the group, Mann–Whitney *U* test and Student’s *t* test were used

As showed in Table 4, the contributions of total drinking fluids to TWI in OH group was the highest and the lowest was in HH group (*P *< 0.001), ranging from 54.1 to 42.6%. Moreover, the lowest contributions of water from food to TWI was in OH group, with the highest was in HH group (*P *< 0.001), ranging from 45.9 to 57.4%. According to the contributions of different fluids to total drinking fluids, water appeared to be the major contributor to total drinking fluids, representing 84.2% in OH group, 80.2% in MH group and 63.5% in HH group (*P *< 0.05). OH and MH groups had 16.7–21.1% higher, 13.3–16% and 1.1–1.8% lower contributions of water, SSBs and alcohols to total drinking fluids than that in HH group, respectively (*P *< 0.05). According to the patterns of water from food, water from dishes contributed 51.0–54.7% to water from food among the three groups and significantly differences were found in the percentages of porridge and snacks in water from food (*P *< 0.001; *P* = 0.004). Both in males and females, the percentages of total drinking fluids and water from food in TWI were higher and lower among participants in OH group than others (*P *< 0.05). In males, the contributions of water to total drinking fluids and the percentages of water from snacks in water from food in OH and MH groups were higher than that in HH group (*P *< 0.05). In females, the contributions of water to total drinking fluids and the percentages of water from porridge in water from food in OH and MH groups were higher than that in HH group (*P *< 0.05). In addition, males had 4.3% lower, 5.4% and 1.1% higher contributions of milk and milk products, SSBs and alcohol to total drinking fluids than females in the three groups (*P* < 0.05).

### Comparison of the urinary and plasma biomarkers

As shown in Table 5, OH group produced 551–950 mL more volume of urine, and excreted a significantly less concentrated urine (less concentrations of K, Na, Cl, Ca, Mg, phosphate, creatinine, uric acid and urea in the urine) than their counterparts in MH and HH groups (*P* < 0.05). The USG were significantly higher in HH group than that in OH and MH groups (*P *< 0.001), ranging from 1.021 to 1.012. Both in males and females, there were significant differences in the urinary biomarkers in the three groups (*P *< 0.05). In plasma biomarkers, as shown in Table 6, only the concentrations of phosphate were different among the three groups (*P* = 0.001). Significant differences were found only in the concentrations of phosphate in males (*P* = 0.035), but no significant differences among females in the three groups (*P > *0.05). Besides, significant differences were found in urinary and plasma biomarkers between males and females in the same group, with males producing a greater urine volume than females, and excreting a significantly lower quantity of solute over each 24 h period (all *P *< 0.05).

**Table 5:** The urinary biomarkers of participants

	OH (n = 58)			MH (n = 72)			HH (n = 26)		
	Male (n = 19)	Female (n = 39)	Total (n = 58)	Male (n = 44)	Female (n = 28)	Total (n = 72)	Male (n = 17)	Female (n = 9)	Total (n = 26)
Volume	1664 (522)*	1740 (732)*	1727 (720)*	1268 (371)^†^	1082 (326)^†^	1176 (345)^†^	853 (188)^‡^	712 (301)^‡^	777 (265)^‡^
Osmolality (mOsm/kg)	430 (86)*	382 (146)*	406 (120)*	685 (141)^†^	616 (158)^†^	662 (156)^†^	879 (132)^‡^	872 (84)^‡^	875 (94)^‡^
USG (urine specific gravity)	1.013 (0.003)*	1.012 (0.003)*	1.012 (0.002)*	1.017 (0.003)^†^	1.015 (0.002)	1.015 (0.003)^†^	1.022 (0.006)^‡^	1.018 (0.002)^‡^	1.021 (0.005)^‡^
pH	6.7 (0.3)	6.8 (0.5)	6.7 (0.5)	6.7 (0.3)	6.8 (0.4)	6.7 (0.3)	6.7 (0.4)	6.8 (0.2)	6.7 (0.3)
Na (mmol/L)	147 (78)*	118 (59)*	124 (55)*	227 (81)	169 (66)	201 (95)^†^	226 (162)	234 (102)	229 (130)^‡^
K (mmol/L)	24.1 (14.8)*	23.9 (10.3)*	24.0 (10.6)*	39.8 (11.8)	34.3 (16.6)^†^	37.8 (14.7)^†^	51.0 (20.5)^‡^	48.8 (11.4)^‡^	48.9 (16.9)^‡^
Cl (mmol/L)	141 (75)*	119 (50)*	121 (51)*	228 (82)	163 (70)	200 (92)^†^	227 (164)	220 (92)	226 (136)^‡^
Ca (mmol/L)	1.54 (1.02)*	1.47 (1.06)*	1.48 (0.93)*	2.43 (1.44)	2.21 (1.19)	2.35 (1.21)^†^	4.70 (2.83)	3.39 (1.94)	3.73 (2.21)^‡^
Mg (mmol/L)	1.77 (1.08)*	1.76 (0.84)*	1.77 (0.91)*	2.73 (1.17)^†^	2.83 (1.02)^†^	2.75 (1.07)^†^	3.37 (1.29)^‡^	3.63 (0.66)^‡^	3.52 (0.94)^‡^
Phosphate (mmol/L)	11.10 (3.46)*	9.63 (4.43)*	10.36 (4.79)*	17.56 (6.94)^†^	14.83 (3.74)^†^	16.63 (6.09)^†^	26.47 (4.40)^‡^	20.46 (9.07)^‡^	25.41 (7.43)^‡^
Creatinine (mmol/L)	7.78 (2.97)*	5.38 (2.29)*	5.95 (2.77)*	11.15 (4.17)^†^	8.88 (2.31)^†^	10.04 (3.60)^†^	15.01 (4.48)^‡^	11.62 (3.79)^‡^	13.79 (4.90)^‡^
Uric acid (mmol/L)	1.63 (0.60)*	1.55 (0.72)*	1.59 (0.66)*	2.80 (0.83)^†^	2.50 (0.69)^†^	2.71 (0.80)^†^	3.70 (1.33)^‡^	3.72 (1.07)^‡^	3.71 (1.25)^‡^
Urea (mmol/L)	161 (69)*	131 (52)*	145 (52)*	258 (86)^†^	193 (81)^†^	231 (92)^†^	337 (82)^‡^	287 (52)^‡^	327 (76)^‡^

Values are shown as the median (M) and quartile ranges (Q)

*There was statistically significant difference between OH and MH groups, *P* < 0.05

^†^There was statistically significant difference between MH and HH groups, *P* < 0.05

^‡^There was statistically significant difference between OH and HH groups, *P* < 0.05. The Kruskal–Wallis *H* test was used to compare the differences among the three groups, including males and females. When comparing the differences between males and females within the group, Mann–Whitney *U* test was used. There were significant differences in the volume of urine, the osmolality, USG, the concentrations of K, Na, Cl, Ca, phosphate, Mg, creatinine, uric acid and urea (*χ*^2^ = 82.291 *P *< 0.001; *χ*^2^ = 131.084, *P *< 0.001; *χ*^2^ = 95.724, *P *< 0.001; *χ*^2^ = 68.277, *P *< 0.001; *χ*^2^ = 62.711, *P *< 0.001; *χ*^2^ = 62.380, *P *< 0.001; *χ*^2^ = 50.843, *P *< 0.001; *χ*^2^ = 90.238, *P *< 0.001; *χ*^2^ = 78.059, *P *< 0.001; *χ*^2^ = 91.976, *P *< 0.001; *χ*^2^ = 98.747, *P *< 0.001; *χ*^2^ = 87.635, *P *< 0.001). In males, significant differences were found in the volume of urine, the osmolality, USG, the concentrations of K, Na, Cl, Ca, phosphate, Mg, creatinine, uric acid and urea among the three groups (*χ*^2^ = 41.540, *P *< 0.001; *χ*^2^ = 64.050, *P *< 0.001; *χ*^2^ = 37.506, *P *< 0.001; *χ*^2^ = 29.684, *P *< 0.001; *χ*^2^ = 24.007, *P *< 0.001; *χ*^2^ = 29.684, *P *< 0.001; *χ*^2^ = 24.892, *P *< 0.001; *χ*^2^ = 23.769, *P *< 0.001; *χ*^2^ = 38.099, *P *< 0.001; *χ*^2^ = 25.223, *P *< 0.001; *χ*^2^ = 38.379, *P *< 0.001; *χ*^2^ = 37.589, *P *< 0.001; *χ*^2^ = 28.930, *P *< 0.001), but there was no significant difference in pH among the three groups (*F* = 2.770, *P* = 0.069). In females, there were also significant differences in the volumes of urine, osmolality, USG, the concentrations of Na, K, Cl, Ca, P, Mg, urea, uric acid and creatinine among the three groups (*χ*^2^ = 45.047, *P *< 0.001; *χ*^2^ = 61.002, *P *< 0.001; *χ*^2^ = 49.910, *P *< 0.001; *χ*^2^ = 30.956, *P *< 0.001; *χ*^2^ = 31.411, *P *< 0.001; *χ*^2^ = 29.374, *P *< 0.001; *χ*^2^ = 20.829, *P *< 0.001; *χ*^2^ = 44.734, *P *< 0.001; *χ*^2^ = 47.734, *P *< 0.001; *χ*^2^ = 46.329, *P *< 0.001; *χ*^2^ = 51.745, *P *< 0.001; *χ*^2^ = 52.709, *P *< 0.001). In OH group, the concentrations of Na, Cl, phosphate, urea and creatinine of urine were different between males and females (*Z* = − 2.245, *P* = 0.025; *Z* = − 2.079, *P* = 0.038; *Z* = − 2.079, *P* = 0.038; *Z* = − 2.725, *P* = 0.006; *Z* = − 4.018, *P *< 0.001). In the MH groups, differences were found in the volumes, the concentrations of Na, Cl, P, urea, USG and creatinine between males and females (*Z* = − 3.038, *P* = 0.002; *Z* = − 3.494, *P *< 0.001; *Z* = − 3.465, *P *< 0.001; *Z* = − 2.645, *P* = 0.008; *Z* = − 3.281, *P* = 0.001; *Z* = − 2.282, *P* = 0.022; *Z* = − 3.823, *P *< 0.001). In HH group, there were significant differences in the concentrations of urea between males and females (*Z* = − 2.398, *P* = 0.016)

**Table 6:** The plasma biomarkers of participants

	OH (n = 58)			MH (n = 72)			HH (n = 26)		
	Male (n = 19)	Female (n = 39)	Total (n = 58)	Male (n = 44)	Female (n = 28)	Total (n = 72)	Male (n = 17)	Female (n = 9)	Total (n = 26)
Osmolality (mOsm/kg)	299 ± 4	300 ± 6	300 ± 6	299 ± 6	298 ± 5	298 ± 5	299 ± 5	301 ± 6	300 ± 5
Na (mmol/L)	142 ± 1	140 ± 1	141 ± 1	141 ± 1	141 ± 2	141 ± 1	141 ± 1	140 ± 1	141 ± 1
K (mmol/L)	4.6 ± 0.4	4.5 ± 0.4	4.6 ± 0.4	4.6 ± 0.4	4.4 ± 0.3	4.5 ± 0.4	4.6 ± 0.4	4.5 ± 0.3	4.6 ± 0.3
Cl (mmol/L)	104 ± 2	104 ± 2	104 ± 2	104 ± 2	104 ± 2	104 ± 2	104 ± 2	104 ± 2	104 ± 2.2
Ca (mmol/L)	2.56 ± 0.04	2.49 ± 0.08	2.52 ± 0.07	2.52 ± 0.08	2.50 ± 0.07	2.51 ± 0.07	2.52 ± 0.05	2.50 ± 0.05	2.51 ± 0.05
Phosphate (mmol/L)	1.31 ± 0.12	1.39 ± 0.16	1.36 ± 0.15*	1.23 ± 0.14	1.34 ± 0.14	1.27 ± 0.15	1.20 ± 0.18	1.37 ± 0.11	1.26 ± 0.18^‡^
Mg (mmol/L)	0.93 ± 0.05	0.91 ± 0.06	0.92 ± 0.05	0.93 ± 0.05	0.90 ± 0.05	0.92 ± 0.05	0.92 ± 0.06	0.91 ± 0.06	0.92 ± 0.06

Values are shown as the mean ± standard deviation (SD)

*There was statistically significant difference between OH and MH groups, *P* < 0.05

^†^There was statistically significant difference between MH and HH groups, *P* < 0.05

^‡^There was statistically significant difference between OH and HH groups, *P* < 0.05. The One-way ANOVA was used to compare the differences among the three groups, including males and females. When comparing the differences between males and females within the group, Student’s *t* test was used. Differences were found in the concentrations of Na (*F* = 7.075, *P* = 0.001). In OH group, the concentrations of Na and Ca were different between males and females (*t* = − 3.411, *P* = 0.001; *t* = − 4.488, *P *< 0.001); in MH group, differences were found in the concentrations of phosphate and Mg (*t* = 3.472*, P* = 0.001; *t* = − 2.427, *P* = 0.018); and in HH group, differences were found in the concentrations of Na and phosphate between males and females (*t* = − 2.849, *P* = 0.009; *t* = 3.050, *P* = 0.006)

## Discussion

There were many factors affecting the water intake of people, including the physiological factors (race, age, gender and physical activity), environmental factors (including season and climate), cultural factors (such as the selection of food intake, cooking methods). Therefore, it was important to record the information of environment in the study areas. Moreover, it was also of vital important to take into account the factors that may influence the intake of water when comparing the results with other studies.

In this study, the TWI and total drinking fluids among the young adults were 2342 mL and 1135 mL, respectively, which were both lower than that of the results of the previous study implemented among adults in four cities in China [8]. Moreover, the results in our study were similar to that of a cross-sectional study conducted among young males [9]. The differences in the age group and the climate may accounted for the differences in the TWI and total drinking fluids. The age groups and season of this study was 18–23 years and winter, respectively, while, that of the survey conducted in the four cities was 18–60 years and summer. It stemmed from our results that there was only 37.1% of the participants in free-living conditions were in optimal hydration status, including 23.8% males and 51.3% females and about 16.7% of them (21.3% males and 11.8% females) were in hypohydration. The results in this study were in accordance with the study investigated among young male adults in China, with only 35.3% of them were in optimal hydration status [9]. The percentage of participants with optimal hydration status was much lower than the results of the study conducted among European adults, in which about 60% of the participants were in optimal hydration status and 20% were hypohydrated [29]. These differences may be due to many factors including the differences in the methods of assessing the hydration status according to the osmolality of urine between this study and the study conducted among three European countries [9, 22, 30, 31]. Hypohydration had been linked with the negative effects on health, especially on cognitive performances. Therefore, it is necessary to raise the awareness of the importance of optimal hydration status for health among young adults. Our results also showed that more females were in optimal hydration status than males in this study. According to the results of the fluids intake among adults from 13 countries, females were more likely to meet the AI of total drinking fluids of EFSA than males [7]. It may be explained by that females maybe more conscious about their health, and it would not be a burden for females to remember to drink water.

The results showed that not only male young adults but also female young adults with optimal hydration had higher amounts of TWI, total drinking fluids than those with middle hydration and hypohydration status, which were in accordance with the results of the study among males in China [9]. Significantly more participants in optimal hydration status met the recommendations of TWI of EFSA, WHO than those in other two hydration statuses, both in males and females. And more participants (including males and females) with optimal hydration met the recommendation of China than participants with other hydration status, ranging from 36.2, 11.1 to 0.0%. The results were the same as the study conducted before among young adults, in which about 54.2% of participants with optimal hydration had enough total drinking fluids according to the reference of China and about 6.8%, 0.0% in the other two hydration groups, respectively [9]. Studies demonstrated that the TWI was strongly positively associated with urine osmolality in adults [9, 32], pregnant and lactating women [33]. Regarding to the contributions of total drinking fluids and water from food to TWI, the better the hydration status, the higher the contributions of total drinking fluids to TWI, the lower the contributions of water from food, both in males and females in this study. The results of the fluids intake among participants from four cities in China showed that people obtained about 40% of TWI from water from food [8]. But only in OH group, the proportions of total drinking fluids (54.1%) to TWI were higher than that of water from food (45.9%), both in males and females. In other words, the TWI of participants in MH and HH groups were mainly from food. This finding suggested that the percentage of TWI attributed to water from food may not be consistent across all the hydration statuses, which were similar with the study [34], in which about 23% and 47% of the TWI were obtained from total drinking fluids among participants with higher TWI and lower TWI, respectively. Researches showed that the differences between TWI were mainly from total drinking fluids among people in free-living conditions [20, 26], which indicated that increase the intake of total drinking fluids may improve the TWI of participants in order to help them to achieve better hydration status. And the interventions in increasing total drinking fluids were demonstrated to be succeeded [35], so the institution should take long-term intervention to increase the intake of total drinking fluids among people.

According to the drinking patterns, we observed that males and females with optimal hydration had higher consumptions and contributions of water to total drinking fluids. The result was similar with the study conducted among young male adults in China [9]. In a study conducted among children, participants with optimal hydration had higher water consumption than hypohydrated participants [36], and the component with water and milk was negatively correlated with urine osmolality [18]. In this study, the contribution of SSBs to total drinking fluids among females with middle hydration status was much higher than that among females with optimal hydration. Though, the contributions of SSBs to total drinking fluids in males were not significantly different in the three groups, but the proportions were ranging from 8.5 to 19.1%. It was showed that regular soda and other fluids was positively correlated with urine osmolality [18]. Moreover, water including tap or bottled water delivered optimal hydration group without adding calories [37], and lower consumers of water and fruit juices showed a higher risk of hypohydration [36]. Additionally, it was demonstrated that drinking more than four coups of plain water was associated with decrease in the risk of new-onset overweight among normal-weight adults [38]. Studies also showed that SSBs increased the risk of type 2 diabetes and weight gain in adults and children, while, plain water was linked with reduced energy consumption and help with the management of weight [39, 40]. Therefore, different types of fluids may have different effects on hydration status and nutrition interventions maybe needed to target this situation, to replace SSBs with water to impact young adults’ health. It can be concluded that not only males but also females with optimal hydration status may have better drinking patterns. Moreover, females maybe have better water intake pattern than males.

As known, with the increase of the amounts of food intake, the water from food increased. In our study, we found strong relationship between the amounts of food intake and amounts of water form food, but no significant association between the amounts of food intake and the percentages of water from food in TWI, which meant that the percentages of water from food in TWI was not affected by the amounts of food intake. This may explain by the factors that the sample size of this study was small (only 156 young adults) and the study was conducted only among young adults aged 18–23 years old. Young adults with different hydration statuses had similar amounts of water from food. Interestingly, among females, the better the hydration status, the higher amounts of water from food was consumed. Meanwhile, males with optimal hydration had similar amounts of water from food with participants in the other two groups, which was different with the study conducted among young male adults before in China [9]. We could conclude that participants in hypohydration with lower TWI would not compensate with the amounts of water from food, especially among young male adults, which may add the risk of hypohydration and impede the health of them. The results showed that the water from food was mainly from dishes and staple food, including the amounts and contributions. In the study among adults in four cities in China, the water from food was also mainly from dishes and staple food [41]. It may be associated with the dietary habitat, in which the plants foods were the main source of foods in China [8]. Among males, the consumptions of water from snacks were different among the three groups, and in females, the water from porridge was higher among participants with optimal hydration than others. Further, the snacks in this study were mainly contributed by fruits, which meant that males with optimal hydration may have more fruits than the hypohydrated males. And the porridge in China was included much water and other nutrients, females with optimal hydration may have a better dietary quality. Study demonstrated that 83% of the men and 96% of the women with high fruit and vegetable intakes were adequately hydrated compared with 60% of men and 81% of women who consumed fewer [42]. It was showed adults with higher total drinking fluids had higher diet quality [43]. What’s more, SSBs consumption was associated with the largest reduction in daily diet quality [44]. Therefore, participants with optimal hydration status may have a better diet quality, which may have positive effect on their health. Furthermore, one study showed that adults aged 19 and older with healthier dietary quality had healthier drinking patterns [45].

Studies showed that there were significant correlations between TWI and 24 h urine biomarkers. For example, TWI was associated positively with the volumes of urine [9] and negatively with osmolality, USG [29], urine sodium, potassium concentrations. In this study, males and females with optimal hydration had higher volumes of urine, less concentrated urine than those in the other two groups, which were consistent with the results of the study [9]. Moreover, males had higher TWI, total drinking fluids, water from food and produced a greater urine volume, excreted a significantly greater quantity of solutes than females with the same hydration status, which were similar with the study [34]. In this study, no significant differences were found between males and females in the plasma osmolality. Researchers investigated the differences of urinary indexes among participants with habitual levels of TWI, they found that plasma osmolality did not response to the changes in TWI [34, 46]. In some instances of hypohydration, plasma osmolality did not track closely with acute changes in urinary indices [47, 48]. This may due to its importance to cardiovascular function, therefore, plasma variables may not be affected until substantial body water had been lost [49]. These results went further to suggest that urine biomarkers were sensitive to the changes of TWI among people in free-living conditions, but not plasma biomarkers, which were the same as the study conducted among healthy sedentary individuals [34]. Research showed that people had less than 1.2L/day total drinking fluids may have increased the levels of copeptin to maintain the homeostasis of fluids [34]. And the copeptin was linked to type 2 diabetes and heart disease [50, 51].

There were some strengths and weakness about our study. The methodologies used to assess the fluids intake included the food frequency questionnaires and food diaries, which may underestimate TWI as they cannot capture all drinking events [52]. In this study, the total drinking fluids were assessed by 7-day 24-h fluid intake questionnaire, which included the details of fluids intake, such as the amounts, types, places, and time. Study demonstrated that 7-day fluid specific diary resulted in a higher estimate of TWI [53]. Moreover, the water from food was assessed by duplicate portion method, which would make the intake of water from food more accurate and avoid the record bias. With the strengths above, there were some weakness about our study. Firstly, more ages of the participants were not investigated; secondly, more plasma biomarkers such as copeptin, were not explored.

## Conclusions

In conclusion, young adults with optimal hydration status had better water intake patterns and less concentrated urine. Females may have better water intake patterns than males. Interventions should be proposed to increase the TWI of young adults, in order to improve their hydration status.

## Data Availability

The datasets used during the current study are available from the corresponding author on reasonable request.

## Abbreviations

OH: Optimal hydration
MH: Middle hydration
HH: Hypohydration
TWI: Total water intake
EFSA: European Food Safety Authority
SSBs: Sugar-sweetened beverages
